# A comparative study of Atlantic salmon chilled in refrigerated seawater versus on ice: from whole fish to cold-smoked fillets

**DOI:** 10.1038/s41598-020-73302-x

**Published:** 2020-10-13

**Authors:** Sherry Stephanie Chan, Bjørn Roth, Flemming Jessen, Trond Løvdal, Anita Nordeng Jakobsen, Jørgen Lerfall

**Affiliations:** 1 Department of Biotechnology and Food Science, Norwegian University of Science and Technology (NTNU), 7491 Trondheim, Norway; 2 Department of Process Technology, Nofima AS, P.O. Box 327, 4002 Stavanger, Norway; 3 National Food Institute, Technical University of Denmark, 2800 Kgs. Lyngby, Denmark

**Keywords:** Industrial microbiology, Biochemistry, Ocean sciences

## Abstract

Water and salt uptake, and water holding capacity (WHC) of whole gutted Atlantic salmon superchilled at sub-zero temperatures in refrigerated seawater (RSW) were compared to traditional ice storage. Following the entire value chain, the whole salmon was further processed, and fillets were either chilled on ice or dry salted and cold-smoked. Changes in quality parameters including colour, texture, enzyme activity and microbial counts were also analyzed for 3 weeks. Our results showed that when fish were removed from the RSW tank after 4 days and further chilled for 3 days, an overall weight gain of 0.7%, salt uptake of 0.3% and higher WHC were observed. In contrast, ice-stored fish had a total weight loss of 1% and steady salt uptake of 0.1%. After filleting, raw fillets from whole fish initially immersed in RSW had better gaping occurrence, softer texture, lower cathepsin B + L activity but higher microbiological growth. Otherwise, there were no differences in drip loss nor colour (L*a*b*) on both raw and smoked fillets from RSW and iced fish. Storage duration significantly affected quality parameters including drip loss, colour, texture, enzyme activity and microbial counts in raw fillets and drip loss, WHC, redness and yellowness in smoked fillets.

## Introduction

Water accounts for 60–70% of total weight in Atlantic salmon (*Salmo salar*). The ability to retain water, known as water holding capacity (WHC), is regarded as one of the most important parameters in preserving fish quality. Most free water that can be easily released lies between the actin and myosin filaments of myofibrils in living or pre-rigor muscles. This water, lost as drip during postmortem storage, is also known as drip loss. Both WHC and drip loss can affect surface appearance and texture, thereby the sensory quality of food^1^. A low WHC is related to postmortem changes in the muscle such as myofibril shrinkage, and a high drip loss is usually related to greater protein denaturation^1,2^. These are undesirable as they lead to greater water and nutrients loss, and directly result in lower salmon quality and sale value. Therefore, maintaining a high WHC and low drip loss is a common aim for fish producers.

Superchilling is a food preservation method where the temperature of fish is kept between traditional chilling and freezing^3^. This slows down autolytic biochemical processes in the muscle and inhibits microbial spoilage, hence prolonging shelf life^4^. One way to achieve superchilling is by storing fish in refrigerated seawater (RSW), and the practice of storing fish under chilled conditions in RSW tanks has been widely used in well-boat industries to store bulk catches of live fish. These tanks are holding systems where the chilling medium of either RSW or brine of the same salinity as seawater (3.5%) is continuously pumped through mechanical chillers.

An unprecedented fish slaughter method has recently been introduced in the aquaculture industry, whereby fish slaughter is directly performed onboard fishing vessels at sea after the fish is pumped, gutted and bled then immediately superchilled at temperatures below 0 °C in RSW tanks during transportation^5^. This novel method condenses the traditional three-stage handling process, where fish are pumped into well-boats and transported to land before processing, into only one. Implementing fish slaughter directly on the vessel eliminates the need for storage and transportation of ice, thereby reducing costs. Therefore, applying the synergistic concept of superchilling and storage in RSW tanks potentially provides an environmentally friendly alternative to traditional chilling on ice, while maintaining a constant low internal fish temperature of below 0 °C at the beginning of the value chain. Since seawater has a higher heat transfer coefficient than ice, it removes heat at a faster rate and maximizes the contact between seawater and fish. Immersing fish in seawater thereby gives a comparative advantage over ice storage, as ice storage can be subjected to delays due to the manual labor involved. In addition, RSW preserves meat freshness by allowing gutted fish to bleed adequately and avoids blood retention in the flesh^6^.

Although the storage life of whole fish in RSW is longer than on ice^7^, this could be limited by water and salt uptake^8^. Usually, a 0.5–1% salt uptake is observed in fish stored in RSW^9^. This uptake may limit the performance of RSW systems and fish might acquire a salty taste and become distasteful, affecting further processing and marketing. The amount of salt absorbed depends on various factors such as size, species, lipid content, physiological state, storage time, temperature and salt content of RSW^10^. Nevertheless, only limited recent studies have been conducted on water and salt uptake of whole salmon in RSW^7,11,12^. A 1985 study on dress chinook salmon by Bronstein et al.^7^ suggests that salmon can only be kept in RSW for less than 4 days and that chilled water systems at 0 °C appears to be more advantageous in terms of microbial quality, but their weight changes were more observable and sensory quality lower than iced fish. These findings are also similar to a 1994 study on pink salmon by Himelbloom et al.^11^.

Despite existing commercial practices of storing live fish in chilled RSW tanks, there are only few studies which examine the new concept of superchilling gutted fish from direct cage slaughter, and how superchilling in RSW affects the water holding properties and other quality parameters of Atlantic salmon throughout the whole value chain. Since this is a new field, there is potential for this method to be widely adapted but there is currently a knowledge gap with little scientific research focusing on the quality of fish using this slaughter method. Therefore, the main objective of this study is to evaluate superchilling in RSW by comparing it to traditional storage on ice of Atlantic salmon. This was done by monitoring water and salt uptake, water holding properties and other quality parameters (colour, texture, microbiology, enzyme activity) from whole salmon to fillets, and further to processed cold-smoked fillets. A small scale RSW tank was constructed in the laboratory to mimic this condition.

## Materials and methods

### Experimental design

On 13th of May 2019, 77 Atlantic salmon (*S. salar*) were obtained from a fish slaughtering facility from the west coast of Norway. Fish were pumped, electrically stunned, automatically slaughtered, gutted and thoroughly bled (temperature: 3.9 ± 1.1 °C, weight: 4.5 ± 0.9 kg, condition factor: 1.4 ± 0.2). An 800-L polyethylene fish chilling tank containing RSW (− 0.60 to − 0.88 °C) was also obtained from the same facility.

**Figure 1:**
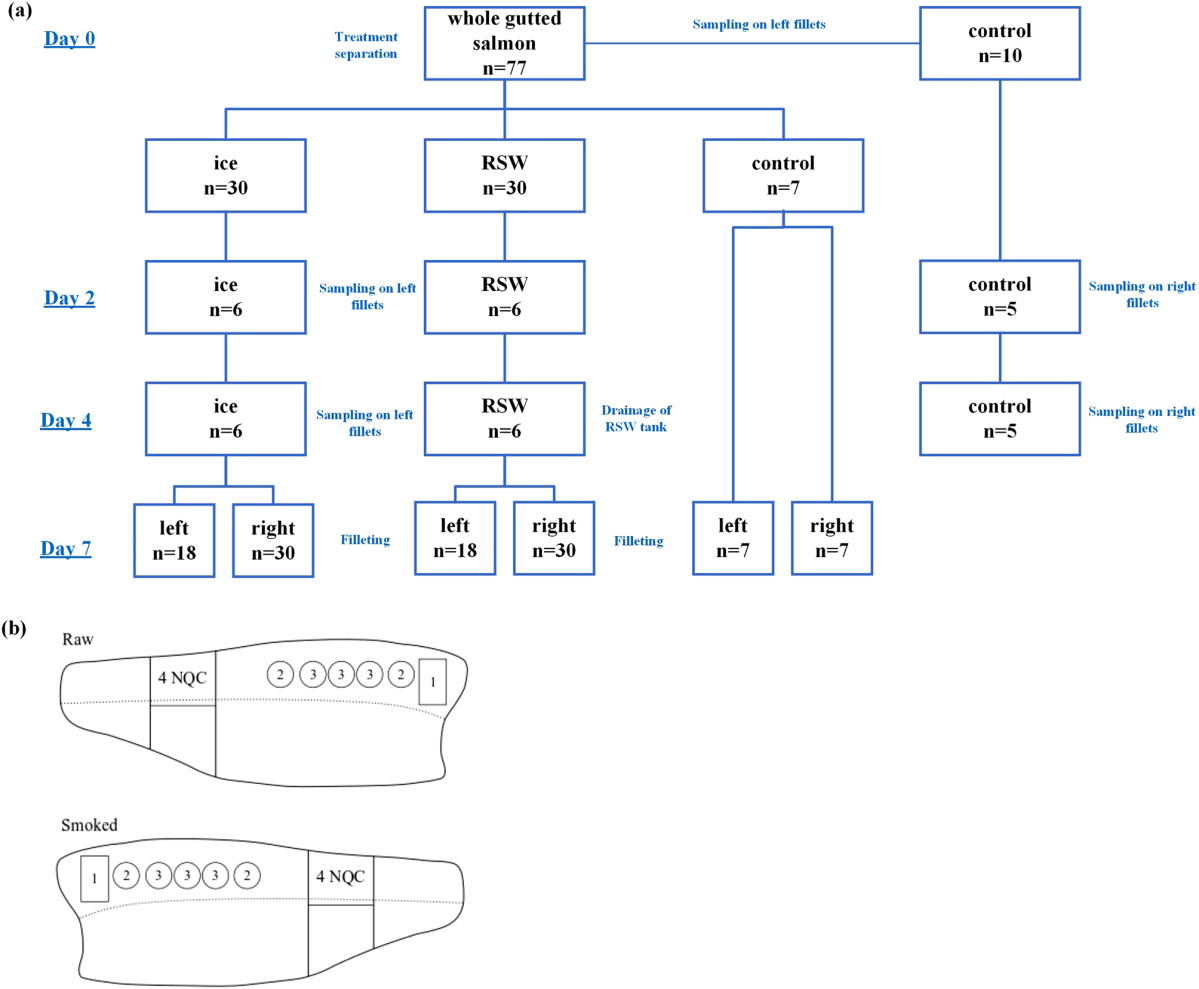
(**a**) Experimental overview, including sample size during storage days. “Control” represents fish wrapped in plastic surrounded by ice, where 10 fish were used to measure WHC on the left fillets from whole fish at t = 0 after slaughter. Thereafter, these fish were kept in a cold room and sampling of WHC was done on the right fillets on days 2 and 4 postmortem. “Ice” represents whole fish stored on ice, “RSW” represents fish stored in refrigerated seawater. “Left” and “right” represents left and right fillets respectively. On day 7 after filleting, left fillets were kept as raw while right fillets were dry salted and cold-smoked. The ice and RSW fillets were sampled periodically for 3 weeks, while control fillets were sampled on the last sampling week. (**b**) Schematic illustration showing weekly sampling locations (n = 6 for raw fillets, n = 10 for smoked fillets). Control fillets were sampled on the last sampling day (n = 7 for raw fillets, n = 7 for smoked fillets). (1) microbiology analysis, (2) frozen samples for enzyme and salt analysis, (3) Water holding capacity and water content, (4) Norwegian Quality Cut (NQC) for texture analysis.

A full factorial experiment was designed (Fig. 1a). Fish were individually tagged and weighed before placing them in either expanded polystyrene (EPS) boxes containing wet ice (n = 30) or in the tank containing RSW (n = 30) prior to a 2 h transport to Nofima AS, Stavanger. 17 fish were used as control, where they were wrapped in plastic then chilled in boxes containing ice, but without any contact with ice. Within this control group, the left fillets of 10 fish were used to determine the initial WHC after slaughter. These fish were then stored before sampling the right side for WHC on days 2 (n = 5) and 4 (n = 5). The remaining 7 fish were filleted on day 7, where left fillets were kept as raw and right fillets were dry salted and cold-smoked.

For each treatment group (ice, RSW, control), two TrackSense Pro temperature loggers (Ellab A/S, Denmark) were inserted in 2 random fish in the mid-abdomen towards the lower back. pH was also measured upon arrival at the laboratory using a Mettler Toledo SevenGo pro pH meter (Mettler Toledo Inc, USA). The tank and EPS boxes were stored in a 0 °C cooling room. Temperature in the tank was constantly monitored and maintained below 0 °C by periodically adding pre-prepared frozen seawater obtained from the International Research Institute of Stavanger (sand filtered, salinity ~ 3.5%). Weights of fish from all groups were recorded on days 2 and 4 in addition to sampling 6 fish from iced and RSW group for WHC, colour and texture analysis. The RSW tank was drained completely on day 4, and fish were gently dried and stored in EPS boxes without ice at 0 °C.

### Processing

On day 7, fish were weighed before being mechanically filleted using a Carnitec fillet machine (Carnitec AS, Støvring, Denmark). The left fillets were stored at 0 °C and weekly sampling was done for quality analysis on 6 fillets from the ice and RSW group (Fig. 1b, t = 7, 15, 22 days postmortem), respectively. On the same day, right fillets were randomized on grids and dry salted with refined salt (GC Rieber, Norway) for 18 h, 0 °C. These fillets were rinsed briefly with cold tap water and gently dried with tissue paper before recording the weight, then cold-smoked using the wood chips protocol of Birkeland and Skåra^13^. The fillets were dried in the smoking chamber for 1 h before smoking and drying 5 consecutive times at alternating intervals of 45 min and 15 min at 22 °C, 75% humidity. They were then cooled, and colour measurements were done on all smoked fillets before vacuum packaging at 99% vacuum and stored at 4 °C. Quality analysis was also done weekly on 10 fillets from the ice and RSW group (t = 16, 23, 29 days postmortem). As for the control group, quality analysis was done on the last sampling day for both raw and smoked fillets (n = 7). Mini temperature loggers (Ellab A/S, Denmark) were placed in boxes containing the fillets from different treatments throughout storage.

### Quality analysis

#### Weight change and water holding capacity

For drip loss calculation, whole fish from all groups were weighed on days 0, 2, 4 and 7. Raw fillets from the ice and RSW group were periodically weighed on t = 7, 15, 22 days while smoked fillets on t = 7, 8, 9, 16, 23, 29 days. Fillets from the control group were weighed on the last sampling day. Drip loss (%) was calculated using the formula ((m_0 _− m_t_)/m_0_) × 100, where m_0_ is the initial weight (g) and m_t_ the weight (g) on the sampling day t.

Water holding capacity (WHC) and water content (WC) were calculated involving an applied force described by Skipnes et al.^14^. Samples were measured in triplicates (diameter 31 mm, height 6 mm) from the dorsal back above the lateral line on the white muscle tissue during sampling days of whole and processed fish (Fig. 1b).

#### Enzyme analysis

Approximately 1 g of frozen sample collected from raw fillets was added in a ratio of 1:5 into the solvent of 0.25 M sucrose solution containing 1 mM EDTA (Sigma-Aldrich, Norway) and 100 mM NaCl (Sigma-Aldrich, Norway) in phosphate buffer (3.38 mM Na_2_HPO_4_ (Merck, Germany), 15 mM NaH_2_PO_4_ (Merck, Germany), pH 7.5). The mixture was homogenized using an Ultra Turrax T25 (Janke and Kunkel IKA, Labortechnik, Staufen, Germany) at 13,500 rpm, 40 s, 4 °C. The homogenates were centrifuged, and supernatants collected for cathepsin B + L analysis using the method of Kirschke et al.^15^. Cathepsin B + L activity was measured in replicates fluorometrically at excitation and emission wavelengths 360 nm and 460 nm respectively, via the release of 7-amino-4-methylcoumarin from substrate Z-Phe-Arg-Nmec.

#### Flesh quality analysis

The extent of fillet gaping was visually recorded according to Andersen^16^ on both raw and smoked fillets on a scale from 0 to 5, where 0 and 5 means no and severe gaping, respectively. For days 2 and 4, right fillets from whole fish used for sampling were manually filleted for colour and texture measurements. Colour analysis was implemented using computer vision with a digital colour measurement system (DigiEye full system, VeriVide Ltd, UK) connected to a SLR (Nikon D80, 35 mm lens, Nikon Corp., Japan). The fillets were placed in a lightbox (daylight, 6400 K) and the images taken were analyzed using the Digipix software (www.verivide.com, version 2.8, VeriVide Ltd., UK) to measure L*a*b* values, where L* represents lightness (L = 0, black; L = 100, white). The a* value changes from − a (greenness) to + a (redness) while b* value changes from − b (blueness) to + b (yellowness)^17^. Colour analysis was also carried out on gills after filleting on day 7.

Texture analysis was measured by a puncture test using a Texture Analyzer TA-XT plus (Stable Micro Systems, UK) equipped with a 12.7 mm flat end cylindrical plunger to poke three consecutive punctures of each muscle sample above the mid-line of the Norwegian Quality Cut (NQC, Fig. 1b). The force (N) was recorded in a texture profile curve operated by the Texture Exponent light software (www.stablemicrosystems.com/, Stable Micro Systems) with a 5 kg load cell at a rate of 2 mm s^−1^ until the probe reached 80% of the fillet height.

#### Salt content

Salt content (% NaCl) was determined with a titration method using SI Analytics Titroline 7000 connected to the software TitriSoft 3.15 and an AgCl 62 electrode (www.xylemanalytics.com/en/, Xylem Analytics, Norway). The solvent and titration agent used were distilled water and 0.1 mol l^−1^ AgNO_3_ (VWR International, Norway) respectively. 100 ml of warm deionized water was added to approximately 2 g of sample. The mixture was then homogenized using an Ultra Turrax T25 (Janke & Kunkel IKA, Labortechnik, Staufen, Germany) at 13,500 rpm for 40 s before adding 1 ml HNO_3_ (VWR International, Norway). Automatic titration was performed, and titration stops at the equivalence point where AgCl is formed. Salt content is calculated using the formula (Eq-B)*T*M*F1/W, where Eq is the volume (ml) of AgNO_3_ consumed at equivalence point; B = 0, blank value; T = 0.1 mol l^−1^, the concentration of titrant; M = 58.44 g mol^−1^, molecular weight of NaCl; F1 = 0.1, conversion factor for % and W = sample weight (g).

#### Microbiological analysis

Muscle pieces (~ 10 g, without skin) were aseptically excised from the anterior part of the epaxial muscle and transferred to a sterile stomacher bag diluted with 1:10 sterile buffered peptone water (Merck, Germany). The mixture was blended using a Smasher (AES Laboratorie, bioMérieux Industry, USA) for 120 s. Homogenates were further diluted to appropriate concentrations. For raw fillets, total psychotropic counts (TPC) were quantified using long and hammer (L&H) agar, while total mesophilic counts (TMC) and H_2_S producing bacteria (HSPB) were quantified using iron agar supplemented with 0.04% l-cysteine (Sigma-Aldrich, Norway) according to the NMKL method No. 184^18^. 49.2 µl of each homogenate was transferred to L&H agar using an Eddy Jet 2 W Spiral Plater (IUL micro, Spain) while 1 ml was transferred to iron agar. L&H agar plates were incubated at 15 °C for 5 days, while iron agar at 25 °C for 72 ± 6 h. HSPB was quantified by the black colonies produced. For smoked samples, microbial analysis was done on the last sampling day using L&H and MRS (de man, Rogosa, Sharpe, Oxoid, UK) agar with Amphotericin B to quantify for TPC and lactic acid bacteria (LAB), respectively. MRS plates were incubated in anaerobic conditions at 25 °C for 5 days according to the NMKL method No. 140^19^. The end of shelf life was defined as the point where microbial counts exceeded 10^6^ cfu g^−1^.

### Statistics

Minitab Version 19 (www.minitab.com, Minitab Inc., USA) were used for all statistical analysis, while Microsoft Visio 2016 (Microsoft Corporation, USA), Inkscape 1.0 (www.inkscape.org) and SigmaPlot 14.9 (www.systatsoftware.com/products/sigmaplot/, Systat Software, USA) were used for figures and plotting of results. Normality tests were assessed based on the normality probability plots. Association among treatment, storage days and response variables were analyzed using general linear model (GLM). Fillet height was added as an additional covariate for texture analysis in GLM. A model including the interaction effect was first tested and the interaction term included when it was significant. Otherwise, a non-interaction model was used. A non-parametric Kruskal–Wallis test was used for comparison of salt content of smoked sampling on the last sampling day, while a one-way ANOVA was used for comparing microbial counts of smoked salmon and gill colour after filleting on day 7. The significance level was set at *p* < 0.05. All results are presented as mean ± standard deviation.

## Results and discussion

### Temperature, water holding properties and salt content

**Figure 2:**
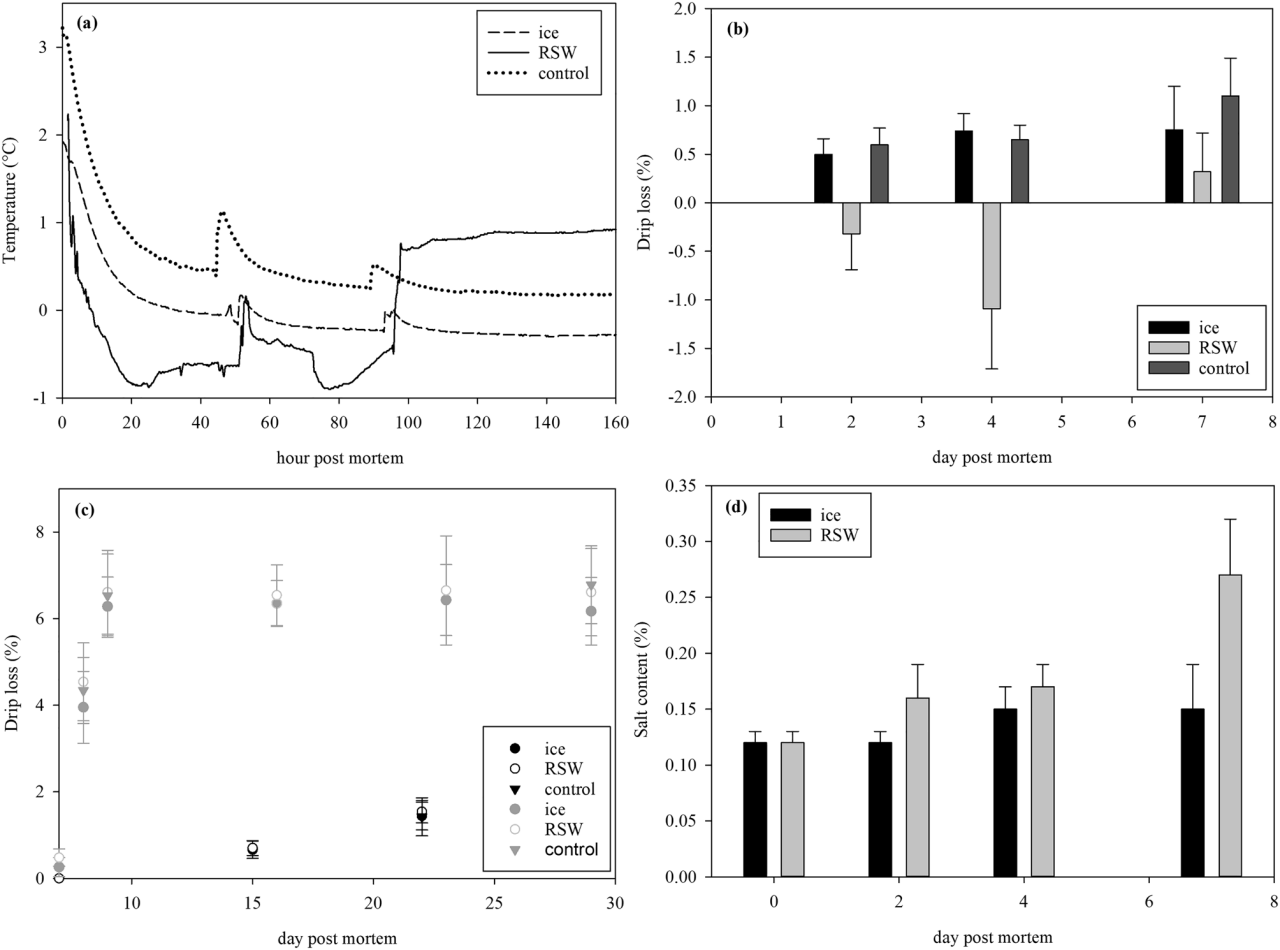
(**a**) Temperature change and (**b**) drip loss of whole fish on ice, RSW and control until day 7 (GLM; storage days: *p* < 0.001; treatment: *p* < 0.001); (**c**) drip loss of raw fillets (black line; GLM; storage days: *p* < 0.001; treatment: *p* = 0.747) and smoked fillets (gray line; GLM; storage days: *p* < 0.001; treatment: *p* = 0.737) from whole fish on ice, RSW and control; (**d**) salt content of whole fish on ice and RSW (GLM; storage days: *p* < 0.001; treatment: *p* = 0.05; days*treatment: *p* < 0.001).

The pH of fish measured 2 h after slaughter was 6.3 ± 0.2. Since a typical pH value for rested fish is around 7.5^6,20^, this indicated that fish used in the present study were likely stressed prior to slaughter and went rapidly into rigor mortis. Roth et al.^6^ and Lerfall et al.^21^ reported that stress from slaughter and pumping can cause a significant decrease in muscle pH. In the experiment, fish were transported from a well-boat to resting cages, crowded then vacuum pumped to the slaughter site. This likely caused the pH decline, suggesting high lactic acid accumulation from glycogen reserves. Internal temperatures of whole fish after 20 h until filleting on day 7 were rather stable for all groups (Fig. 2a). The observed fluctuation and rise in temperatures at 48 h and 96 h were due to the need for weighing and sampling. Removal of fish from RSW tank also caused the temperature increase at 96 h for RSW fish.

Temperatures of raw and smoked fillets for all groups were kept stable during storage at 0 °C and 4 °C respectively (data not shown). It is established that temperature during superchilling must be kept as stable as possible to prevent repeated ice recrystallisation and prolong the product’s shelf life^12^. In the present study, it was inevitable to disrupt the cold chain even only for a short period during sampling days. However, temperature is usually maintained rather constant during storage in a commercial scale so this should not greatly affect shelf life.

There was a significant effect of storage days and treatments on drip loss in whole fish (Fig. 2b, *p* < 0.001, *p* < 0.001, respectively). The drip loss of iced fish stabilized at around 1% while those of control fish gradually increased during the 4 days of storage. Similarly, Erikson et al.^12^ reported that ice-stored salmon lost weight during the first 2 days before stabilising at a drip loss of 2%. The present study illustrates that RSW fish gained 1% seawater by day 4. After seawater was drained on day 4, RSW fish lost 0.3% in drip with reference to its initial weight during the additional 3 days of storage. Taking into account that the weight gained on day 4 was greater than weight lost on day 7 with respect to its initial weight, the overall weight gain after 7 days of storage for RSW fish was 0.7%. Previous studies have reported a higher weight gain of salmon kept in RSW for 4 days. Erikson et al.^12^ reported a 2.5% gain in gutted Atlantic salmon kept in − 2 °C, while there was a 3% gain when pink salmon was stored in − 0.5 °C^11^. Bronstein et al.^7^ measured a 2% gain at 0 °C. Based on these studies, the chilling temperature seemed to have minimal effect on weight gained in fish. Gutting fish before immersion in RSW results in water and salt uptake through the abdominal cavity, causing weight gain^12^. The comparatively lower gain observed in this experiment could be due to the larger salmon size used which renders a slower diffusion of water into the muscle. Differences in fat content of various salmon species reported may have also affected water and salt uptake.

There was an effect of storage day (*p* < 0.001) but not of treatment (*p* = 0.747) on drip loss in raw fillets (Fig. 2c). Drip loss increased steadily to 1.4–1.5% for all three groups. The product yield after smoking for all fillets was 94% and there was no effect of treatment observed (*p* = 0.737). Drip loss of smoked fillets increased to 6.5% after smoking for all groups and plateaued throughout storage. There was also no difference in salt contents at the end of storage (*p* = 0.733; RSW: 3.2 ± 0.4%, ice: 3.1 ± 0.7%, control: 3.0 ± 0.2%). The large increase in drip loss before and after dry salting and cold-smoking was mainly due to the diffusion and evaporation of water from the muscle to the surface. Since the drip loss of fillets from all treatments were similar, the observed increase in drip loss was only affected by storage duration (*p* < 0.001). Therefore, the choice of storage regime on whole fish seems unlikely to affect the drip loss of cold-smoked fillets. Comparison of salt uptake indicated that iced fish had a consistent salt content at 0.1%, while RSW held fish almost doubled from 0.1 to 0.2% during 4 days of storage, and further gained to 0.3% after RSW removal (Fig. 2d, storage days: *p* < 0.001, treatment: *p* = 0.05). The effect of storage duration was also significantly influenced by the treatment method (interaction: *p* < 0.001). The results consonates with other studies where chinook salmon had a salt content of 1.1% after 7 days in RSW and 0.1% on ice^7^. Himelbloom et al.^11^ further showed that salt content of pink salmon stored in chilled seawater (CSW) increased to 0.5% during 10 days of storage, while iced salmon maintained at 0.1%. In RSW systems, the addition of salt in water causes the structure of pure water to be disrupted as salt dissociates into Na^+^ and Cl^−^ ions, increasing the ion–dipole interaction between salt and water. The salt gained on day 7 observed in the present study after the removal of RSW possibly signifies that the retained Na^+^ and Cl^−^ ions from seawater that was absorbed continued to diffuse into the fish muscle and binded with the muscle proteins. As such, the drip lost after removal of fish from RSW probably contains mainly water, likely from the free water located outside the myofibrillar network that can be easily lost from the tissue. Nevertheless, the application of salt uptake in Atlantic salmon has been considered relatively unimportant due to its large size and subcutaneous fat layer which hinders salt migration^22^. Hence salt uptake during RSW storage is not considered a problem as it is also dependent on other factors like species, lipid and salt content, temperature, physiological state and storage duration^10^.

**Figure 3:**
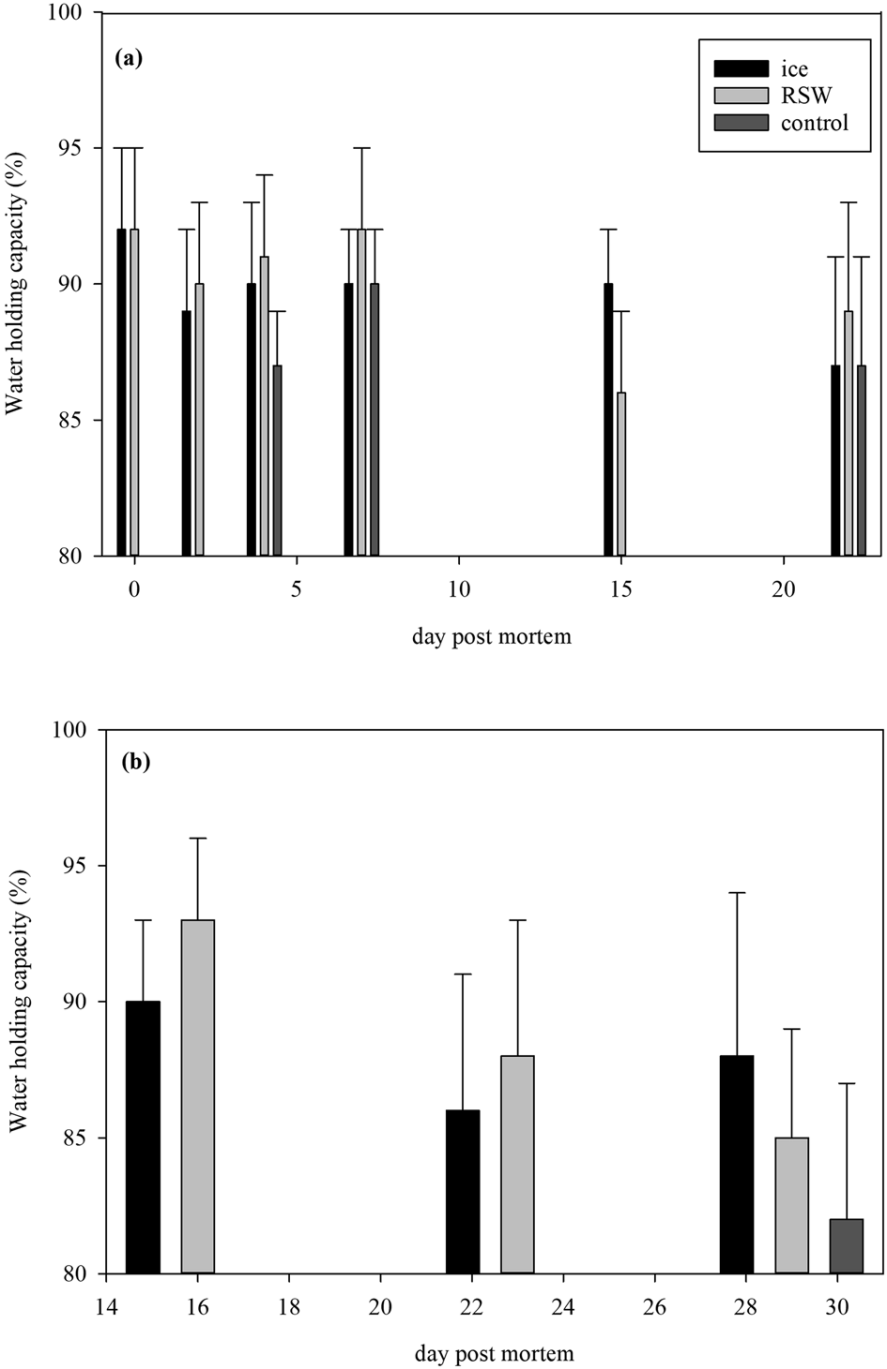
(**a**) WHC (%) of whole fish on ice, RSW and control until day 7 (GLM; storage days: *p* = 0.002; treatment: *p* < 0.001), and after filleting until day 22 (GLM; storage days: *p* = 0.728; treatment: *p* = 0.032); (**b**) WHC (%) of smoked fish from ice, RSW and control until day 29 (GLM; storage days: *p* < 0.001; treatment: *p* = 0.002).

RSW fish had a notably higher WHC than iced and control fish (*p* < 0.001) until day 7 prior to filleting, with also a significant effect of storage days (*p* = 0.002, Fig. 3a). After filleting, there was no effect of storage duration on raw fillets (*p* = 0.728) and no clear pattern was observed among treatments, although statistical analysis revealed a difference (*p* = 0.032). In contrast, there was a general decrease in WHC through storage days for smoked fillets (*p* < 0.001). The control fish had the lowest WHC on day 29, while RSW fish had the highest WHC on days 16 and 23 (Fig. 3b).

Storage of fish in RSW is a brining method where fish is immersed in a 3.5% salt solution. Since the surrounding brine has a higher concentration than the intracellular fluid in the muscle, this results in salt entering the muscle by diffusion, thereby increasing yield. Degree of muscle swelling depends on the salt concentration where maximum swelling and WHC occurs at a 5–6%^23,24^. Previous studies reported that brining allows proteins to retain more moisture, as lower salt concentrations causes a lower degree of protein denaturation and induces swelling of muscle fibers, leading to a higher WHC^25,26^. This occurs due to the repulsive electrostatic forces of Cl^−^ anions weakly attached to the myofibrillar and sarcoplasmic proteins, causing protein to coagulate and entrap free water^25^. At higher salt concentrations, protein denaturation increases and myofibrillar proteins lose water, causing muscle dehydration and eventually yield loss^23^. Storing fish in RSW could therefore be beneficial in moisture retention and potentially give the product a better cooking yield and tenderness. Further studies such as sensory analysis could be done to examine and relate this with industries’ and consumers’ preferences.

### Surface appearance and texture

Lightness (L*) of fillets from whole fish decreased while yellowness (b*) increased until day 4. This is likely due to the flesh of fish becoming less translucent (more opaque), affecting the light absorption and reflection after slaughter of fresh fish^20,27^. The opposite was observed after filleting from days 7 to 15, where L* increased throughout fillet storage and became paler before leveling out (*p* = 0.001, Table 1). A similar phenomenon was observed by Erikson and Misimi^20^, where the L* of ice chilled salmon fillets was darker during the first 20 h and then rose sharply afterwards. This could be influenced by the duration of rigor, as muscle contraction may cause differences in lightness^20^. Redness of raw fillets observed in this study decreased throughout storage (*p* < 0.001). Loss of fillet transparency and redness has been correlated with protein denaturation^28^ and higher liquid loss^27^, in line with observations seen after filleting. Previous studies also revealed that fillets resulted in a lighter, less reddish and yellowish colour in continuous ice storage for 7 days^20^ which tallies to our results for iced fish after filleting.

**Table 1:** L*, a*, b* and 80% compression force of raw and smoked fillets throughout storage.

Group	Raw fillets						Smoked fillets					
	Day	L*	a*	b*	80% compression force (N)	n	Day	L*	a*	b*	80% compression force (N)	n
Ice	2	63.3 ± 2.9	40.6 ± 1.6	30.9 ± 1.6	31.2 ± 5.3	5	9	54.4 ± 1.6	32.9 ± 1.9	34.9 ± 1.3	–	30
	4	56.8 ± 3.6	37.3 ± 1.6	32.9 ± 1.6	28.0 ± 4.8	6	16	53.8 ± 1.8	30.7 ± 1.3	35.7 ± 1.3	26.6 ± 4.6	10
	7	60.1 ± 2.7	34.5 ± 2.6	29.0 ± 2.0	24.1 ± 6.3	6	23	54.0 ± 0.7	29.6 ± 1.8	35.0 ± 1.0	24.6 ± 5.6	10
	15	65.3 ± 1.9	33.1 ± 2.1	29.7 ± 1.9	17.9 ± 4.1	6	29	53.3 ± 2.2	29.4 ± 1.6	33.3 ± 0.8	27.4 ± 6.6	10
	22	65.2 ± 1.6	29.6 ± 1.8	26.9 ± 1.5	17.2 ± 4.5	6						
RSW	2	66.4 ± 4.7	37.4 ± 2.1	28.9 ± 1.6	29.0 ± 6.6	6	9	54.1 ± 2.5	33.1 ± 2.1	34.6 ± 1.5	–	30
	4	58.7 ± 2.7	35.2 ± 2.5	31.7 ± 1.6	24.8 ± 7.7	6	16	54.6 ± 2.5	30.0 ± 1.8	34.5 ± 1.4	28.0 ± 4.4	10
	7	60.5 ± 1.5	37.5 ± 1.5	28.2 ± 1.3	19.8 ± 5.2	6	23	53.5 ± 2.2	30.0 ± 2.6	34.3 ± 1.4	24.0 ± 6.2	9
	15	65.9 ± 2.7	31.4 ± 3.5	28.4 ± 2.0	17.0 ± 4.1	6	29	53.0 ± 1.9	30.3 ± 0.9	33.7 ± 1.4	24.9 ± 5.9	10
	22	64.2 ± 1.4	31.6 ± 0.9	27.7 ± 0.7	19.0 ± 3.8	6						
Control	22	65.8 ± 1.7	29.7 ± 1.9	26.5 ± 1.2	18.7 ± 5.2	7	9	54.7 ± 1.9	33.3 ± 1.8	35.4 ± 1.7	–	7
							29	54.6 ± 1.8	27.8 ± 1.51	33.9 ± 1.0	24.3 ± 5.6	7
GLM^a^	P_D_	0.001*	< 0.001*	< 0.001*	< 0.001*		P_D_	0.057	< 0.001*	0.001*	0.120	
	P_G_	0.478	0.823	0.106	0.006*		P_G_	0.334	0.689	0.254	0.635	
	P_H_	–	–	–	0.001*		P_H_	–	–	–	< 0.001*	

^a^General Linear Model (GLM) analyses of variance with fillet groups as factors and storage days as covariance. Fillet height was added as an extra covariate for texture analysis. P_D_, P_G_ and P_H_ are the significant levels for the effects of the storage days, groups and fillet height, respectively.

*Significant when *p* < 0.05.

There was no effect of treatment on the colour of raw fillets (L*: *p* = 0.478; a* *p* = 0.823; b*: *p* = 0.106). The RSW fish were slightly lighter and less yellowish in colour than iced. A common problem associated with RSW stored fish is the bleaching of fillets which may hinder its market value^10^. Bleaching was not seen in the fillets, as adjacent to the findings of Erikson et al.^12^ who found that continuous storage of fish in RSW did not lead to lighter fillets. By visual observation, gill colour from the RSW fish seemed to be grayer and less reddish in colour which could be a more obvious evidence of bleaching, as also reported in RSW-stored cod for 4 days^29^ and RSW-stored ocean pearch for several days^30^. This was further verified by the present study when the lightness observed on day 7 for the RSW fish was significatly higher (*p* = 0.001; ice: 36.2 ± 3.2; RSW: 41.1 ± 3.4; control: 39.4 ± 3.2), while redness lower (*p* = 0.001; ice: 18.1 ± 3.2; RSW: 13.2 ± 2.5; control: 16.0 ± 4.4) than both the iced and control fish. Nevertheless, quality of fillets from RSW stored fish are still considered highly acceptable with its shelf life surpassing traditionally iced fish^31^.

The smoked fillets from all treatments had a decrease in redness and yellowness through storage (a*, *p* < 0.001; b*, *p* = 0.001), while no differences were observed among treatments (L*, *p* = 0.334; a*, *p* = 0.689; b*, *p* = 0.254). As accurately seen in this study, a general trend in cold-smoked salmon is that they are darker and less red, but more yellowish than the unprocessed fillets^32^. Dry salting is a process that affects colour and texture, causes cell shrinkage and decreases the thermal stability of actin and myosin which eventually leads to protein denaturation within the muscle. Limited reports are currently available on colour changes in both raw and smoked fillet as affected by RSW storage. However, results from the present study as well as visual observations indicated that the colour quality of raw and smoked fillets from RSW fish are comparable to those on ice.

The puncture test showed that texture of raw fillets decreased throughout storage (*p* < 0.001), an established fact of muscle tenderization due to the gradual disintegration of connective tissues^5,33^. Afterwards, the divergence becomes minimal and gradually stabilizes through storage. The compression force was significantly affected by the fillet height (*p* = 0.001). Storing and removal of fish in RSW also produced softer fillets as they had a significantly softer texture in comparison to iced fillets (*p* = 0.006). This was attested by Erikson et al.^12^ and Chan et al.^5^ who asserted that changing the chilling medium from RSW to storage with or without ice leads to significantly lower hardness. One would therefore expect that the fillets of RSW fish will cause a higher gaping incidence. Gaping is a damaging textural problem causing flesh softening from the collapse of muscle and collagen fibrils^16,34^. In the present study, the extent of gaping for the ice-stored and control raw fillets increased through time (*p* < 0.001), coinciding with the decrease in compression force. Interestingly, there was almost no gaping occurrence for raw fillets from RSW fish on days 7 (ice: 0.5 ± 0.5; RSW: 0.0 ± 0.2) and 15 (ice: 1.0 ± 0.3; RSW: 0.0 ± 0.0; control: 3.0 ± 0.7). This suggests that softness might be due to other factors apart from fibral disintegration. It should also be noted that texture and gaping in fish can be influenced by a variety of factors such as harvest season, body size, collagen composition and water content^34^.

There was no difference in textural quality among the smoked products neither in storage day (*p* = 0.120) nor treatment per se (*p* = 0.635), while the compression force was significantly affected by the fillet height (*p* < 0.001). In terms of gaping, the control fish had the highest gaping score on the last sampling day (ice: 1.0 ± 0.7; RSW: 1.0 ± 1.0; control: 2.0 ± 1.1). Therefore, the effect of treatment method on both texture and gaping seemed to apply only to the unprocessed fillets in this experiment. Hansen et al.^35^ stated that textural deterioration occurs in cold-smoked salmon due to autolytic deterioration of tissue which develops rancid and oxidised off-flavours. However, the decline in textural properties for all smoked salmon groups was not apparent in our study, and is in agreement with earlier studies which found that shear force of cold-smoked salmon was stable during cold storage^36,37^.

### Enzyme and microbial activity

**Figure 4:**
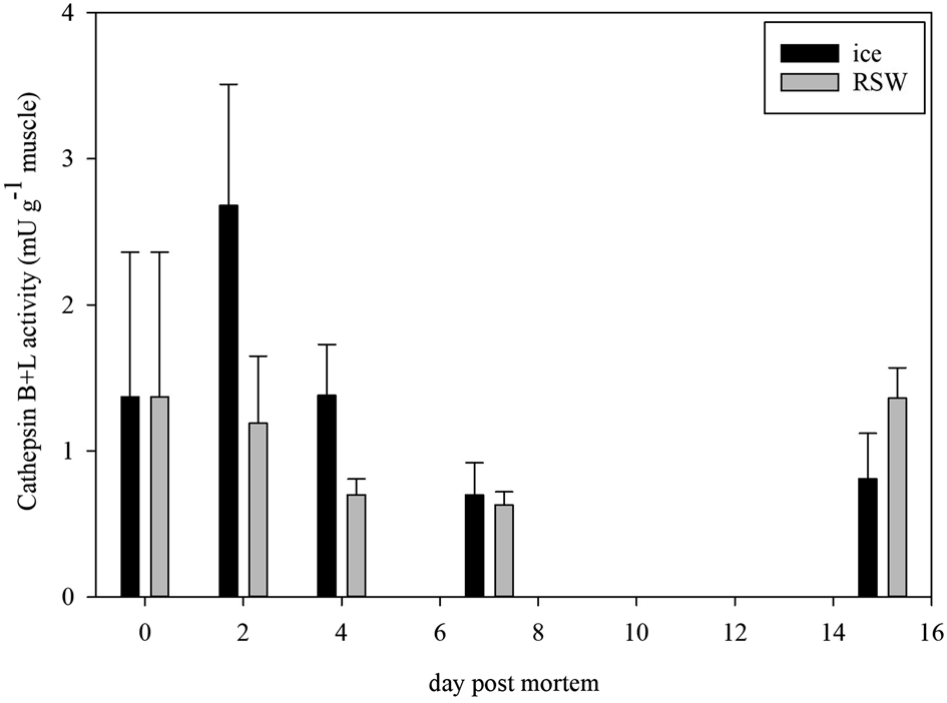
Cathepsin B + L activity of ice and RSW fish (n = 5; GLM; storage days: *p* = 0.035; treatment: *p* < 0.001; days * treatment: *p* = 0.002).

The lysosomal cathepsins B + L are proteases believed to degrade muscle collagen which causes tissue softening and has been used to explain the degree of proteolysis^38,39^. In the present study, significant differences in muscle cathepsin B + L activity were observed between the iced and RSW fish. Samples from whole iced fish had a consistently higher enzyme activity from days 2 to 7 (Fig. 4, *p* < 0.001) and decreased through storage (*p* = 0.035), while those from RSW fish increased in enzyme activity on day 15. There was also an interaction effect between storage duration and treatment (*p* = 0.002).

Temperature is a main determinant in both enzyme and microbiological activity. The enzyme activity observed in RSW fish during the first 7 days is possibly explained by a lower refrigerated temperature when fish were kept in RSW, suppressing the activity. When RSW fish were kept at the same temperature conditions as iced fish afterwards, the enzyme activity increased on day 15. Although whole fish from RSW resulted in a softer texture than iced fish before they were filleted, the enzyme activity apparently does not reflect this. A plausible explanation for the softer texture of RSW fish could be due to its water and salt uptake during immersion in seawater. Contrary to this, the observed softening in iced fish was likely due to increased enzyme activity during chilled storage especially during the first 48 h. Gaarder et al.^40^ stated that cathepsin B + L activity increases to a threshold until 24 h postmortem and remains stable afterwards, while Duun^2^ presented that the activity was stable during ice storage, indicating that these enzymes were still active and led to softening during storage. Based on our knowledge, the enzymatic activity including other enzymatic reactions involved in postmortem softening of tissue like collagenases and calpains from fish stored in RSW has not been thoroughly explored. This could be an interesting aspect for further studies which can include fish histology to identify the development of intra- and extracellular cell structures during RSW storage.

**Figure 5:**
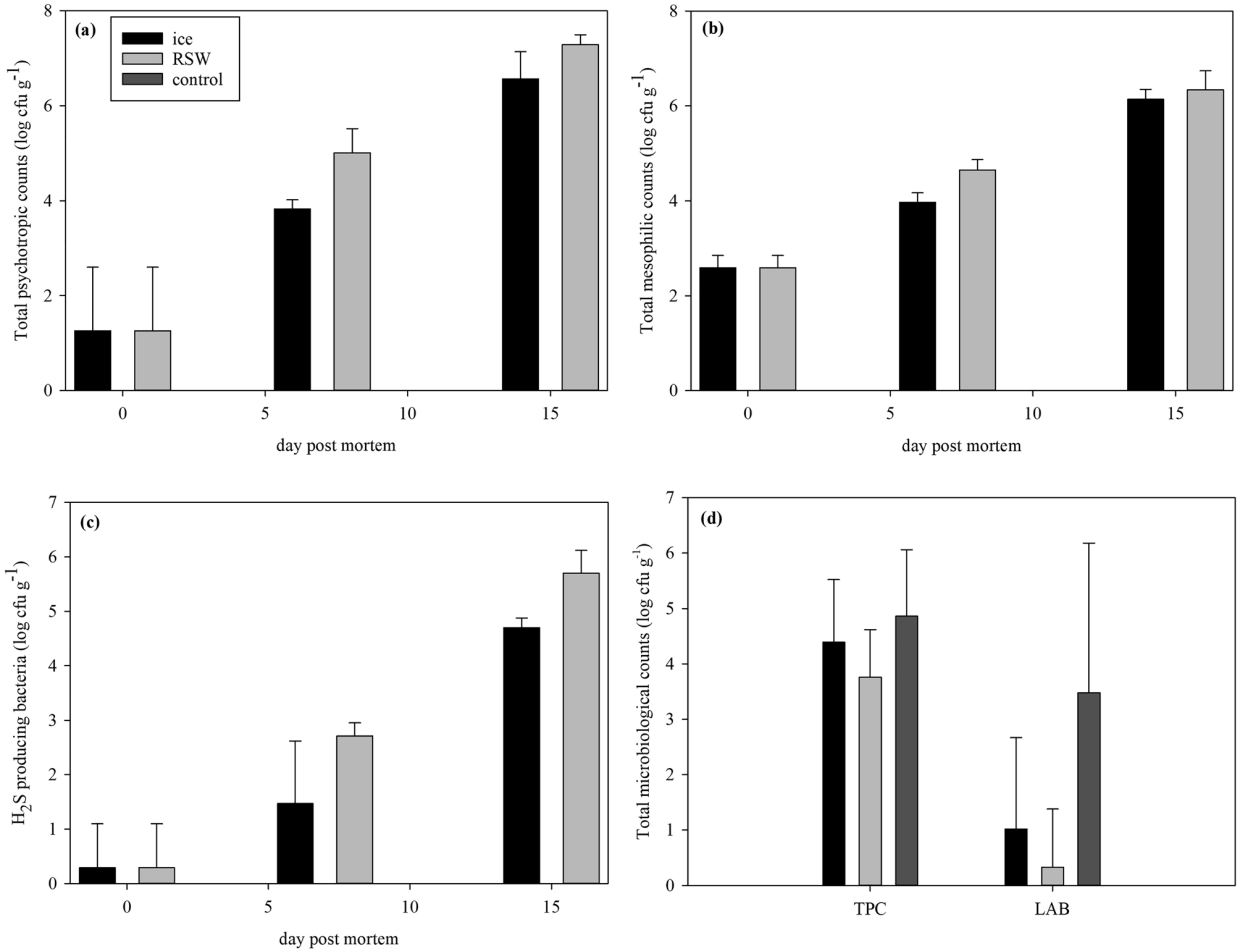
(**a**) Total psychotropic counts of raw fillets from ice and RSW through storage (n = 6; GLM; storage days: *p* < 0.001; treatment: *p* < 0.001); (**b**) Total mesophilic counts of raw fillets from ice and RSW through storage (n = 6; GLM; storage days: *p* < 0.001; treatment: *p* = 0.046); (**c**) H_2_S producing bacteria counts of raw fillets from ice and RSW through storage (n = 6; GLM; storage days: *p* < 0.001; treatment: *p* < 0.001); (**d**) Total psychotropic counts (n = 10; One-way ANOVA, *p* = 0.123) and lactic acid bacteria counts (*p* = 0.005) of smoked fillets from ice, RSW and control treatment on last sampling day.

Microbial counts (TPC, TMC, HSPB) significantly increased throughout storage for both raw fillets from iced and RSW fish (Fig. 5a–c; TPC: *p* < 0.001, TMC: *p* < 0.001. HSPB: *p* < 0.001). Psychotropic and mesophilic bacterial species that can be present in salmon include *Shewanella* spp., LAB, *Photobacterium* spp., *Pseudomonas* spp. and *Brochothrix thermosphacta*^41^. A bacterial prevalence of > 10^6^ cfu g^−1^ depicts the end of shelf life^35,42^ so under this criteria both treatments were deemed spoiled after 15 days. RSW fish had a significantly higher counts on TPC (*p* < 0.001), TMC (*p* = 0.046) and HSPB (*p* < 0.001). This occurrence was presumably due to a variety of reasons such as challenges of recirculating seawater in the simulated tank. Despite ensuring proper hygiene when handling the fish from the tank, it was still an enclosed system with no recirculation of new seawater. In addition, contamination is a big risk and cross-contamination might have occurred as other experiments were ongoing concurrently when the tank was stored in the cold room. Insufficient cleaning of the RSW tank might have also contributed to the increased microbial growth. Prior to the experiment, the tank was cleaned with foam instead of using a strong detergent like lye, so thorough cleaning could have been challenging for this makeshift tank despite it being kept constant at sub-zero temperatures during the storage period.

Contradictory results have been reported when fish were stored in RSW and on ice possibly due to the difference between laboratory and industrial based experimental scale^43^. The initial microbiological activity observed right after slaughter further suggested that the fish might have blood remnants remaining in the flesh after being bled in the bleeding tanks at the land-based slaughtering facility. The addition of seawater and seawater ice into the tank was also manually done as compared to mechanical procedures in commercial settings. Hence the rate of spoilage for the RSW fish was more pronounced in this study which affirms how easy contamination can occur. However, these problems should not arise in commercial settings due to more stringent rules with regards to filtering, ozonating and chlorinating of process water. Industries also use CO_2_-based RSW tanks which improves energy efficiency, suppresses bacterial growth, and enhances sensory attributes of fish^8^. Good hygiene has been posed as a possible challenge posed by closed RSW tanks as spoilage of fish may affect the entire catch^44^. Therefore, a well-designed RSW system is important and proper considerations must be implemented including good insulation of tanks, evenly distributed and controlled temperatures, constant supply of clean seawater and adequate cleaning and disinfection of the factory after every harvest^10^. This ensures good recirculation of water, lessens microbial spoilage, maintains good quality and offers the flexibility for fishing vessels to travel to further distances.

TPC for RSW treated fish after cold-smoking tended to be lower than both chilled and control fish (*p* = 0.123), while a significantly higher LAB was produced for the control fish (Fig. 5d, *p* = 0.005). The shelf life of vacuum-packed whole fillet cold-smoked salmon at 5 °C was previously reported to be around 32–49 days^35^, while the acceptable shelf life for commercial industries is 21–60 days^45^. LAB is prevalent in smoked salmon, producing organic acids and ethanol as fermentation products, hence off-flavours and off-odours associated with spoilage^35,46^. Hansen et al.^35^ further concluded that spoilage of vacuum packed cold-smoked salmon is due to microbiological activity combined with the autolytic deterioration of tissue, causing textural damage through storage. The actual shelf life of salmon was difficult to conclude in our study with only 3 sampling points conducted. Future considerations may include more sampling points and characterization of other prevalent spoilage microorganisms such as *Pseudomonas* spp. or *Photobacterium* spp. which may be a better microbiological indicator of shelf life^41^. Nevertheless, salting and smoking of salmon is a well-established method to prolong shelf life of fish and it is recommended to further process salmon fillets at early stages of the value chain.

## Conclusion

This study presented several quality parameters examined on salmon stored in RSW throughout the whole supply chain. In comparison to traditional chilling methods, whole fish stored in RSW had an overall increase in water and salt uptake, with better WHC before filleting. After filleting, better gaping scores, softer texture, lower cathepsin B + L activity and higher microbiological growth were observed. Although the raw fillets from RSW fish had a softer texture, this was likely unaffected by the enzymatic process of cathepsins B + L causing postmortem degradation. The microbiological analysis on raw fillets suggested that RSW fish had a shorter shelf life, but this is not representative of commercial practices due to the experimental scale. Drip loss and colour of both raw and smoked fillets from the 2 treatments were comparable, and storage duration was the main determinant affecting these parameters. These results indicate that RSW-stored fish is a viable method in minimizing the need for ice storage and land transportation, thereby introducing economical benefits and contributing to a positive impact on the environment. The idea of shifting fish slaughter from land to sea further introduces several advantages including reduced transportation costs, reduced fish diseases and mortality, increased slaughtering capacity and improved fish welfare. Therefore, the cutting-edge concept of slaughter vessels can provide great potential to increase its competitive advantage in the salmon industry. Industries seeking to understand more about the quality changes during storage of fish in RSW tanks, how this differs from the traditional chilling method on ice and how this affects fillet quality after primary and secondary processing to cold-smoked fillets can consider the results of this study during the formularization and streamlining of their processes.

As temperature is a critical aspect in superchilling, it is crucial to maintain a constant sub-zero temperature during storage combined with proper recirculation and good hygiene practices of RSW systems to lower the risk of contaminating the whole catch. Since this is a relatively new concept, more comprehensive research like shelf life and consumer acceptance studies can be performed to explore more potentials and solutions for such vessels.

## Data Availability

The datasets generated during/or analysed during the current study are available from the corresponding author on reasonable request.
